# Investigating a neural language model’s replicability of psycholinguistic experiments: A case study of NPI licensing

**DOI:** 10.3389/fpsyg.2023.937656

**Published:** 2023-02-23

**Authors:** Unsub Shin, Eunkyung Yi, Sanghoun Song

**Affiliations:** ^1^Department of Linguistics, Korea University, Seoul, Republic of Korea; ^2^Department of English Education, Ewha Womans University, Seoul, Republic of Korea

**Keywords:** neural language model, BERT, negative polarity items, NPI licensing, grammatical illusion, licensing strength, scale of negativity, psycholinguistics

## Abstract

The recent success of deep learning neural language models such as Bidirectional Encoder Representations from Transformers (BERT) has brought innovations to computational language research. The present study explores the possibility of using a language model in investigating human language processes, based on the case study of negative polarity items (NPIs). We first conducted an experiment with BERT to examine whether the model successfully captures the hierarchical structural relationship between an NPI and its licensor and whether it may lead to an error analogous to the grammatical illusion shown in the psycholinguistic experiment (Experiment 1). We also investigated whether the language model can capture the fine-grained semantic properties of NPI licensors and discriminate their subtle differences on the scale of licensing strengths (Experiment 2). The results of the two experiments suggest that overall, the neural language model is highly sensitive to both syntactic and semantic constraints in NPI processing. The model’s processing patterns and sensitivities are shown to be very close to humans, suggesting their role as a research tool or object in the study of language.

## 1. Introduction

For decades, computational modeling has been extensively used in many areas of language research. Some of them are highly concerned with behavioral and biological properties that speakers exhibit during online sentence processing. For example, researchers in natural language processing and computational psycholinguistics have developed and refined computational models that reflect the mechanisms of language processing and produce human-like language output (Trueswell et al., 1994; Jurafsky, 1996; Hale, 2001; Levy et al., 2009; McRae and Matsuki, 2013; Smith and Levy, 2013; Linzen et al., 2016; Caliskan et al., 2017; Lau et al., 2017). In a relatively new field referred to as computational neurolinguistics, researchers attempt to model the direct link between linguistic features and biological bases in the brain (Arbib and Caplan, 1979; Hagoort, 2003; Beim Graben et al., 2008; Huyck, 2009; Beim Graben and Drenhaus, 2012; Barrès et al., 2013; Rabovsky and McRae, 2014; Frank et al., 2015; Brouwer and Crocker, 2017; Carmantini et al., 2017; Venhuizen et al., 2019; Brouwer et al., 2021). The recent advancement in computational modeling of language based on *deep* neural networks further adds innovations to those computationally oriented areas. Due to its architecture, it also provides new insights and research opportunities to other traditional areas of language research such as theoretical linguistics and psycholinguistics.

Current deep neural language models (henceforth, LMs) are built upon large amounts of naturally occurring language data without any information regarding abstract representations or theoretical constructs that linguists have shown to be essential to process the underlying structure of a language. Thus, in terms of the input, LMs “learn” a language in the way average people acquire their first language, i.e., only by experiencing language use without any explicit training in parse trees and ontologies of words. This raises some important questions not only for computational linguists but also for theoretical linguists and psycholinguists. For example, what linguistic knowledge do LMs ultimately derive from the input language? Is it similar to the knowledge that human speakers have about their language? From the viewpoint of processing, is the way the *neural* language processor works similar to the way the *human* processor works? What are the implications of the neural processor’s behavior regarding the notion of grammar and grammatical knowledge?

Some studies have attempted to address these issues by performing analytic evaluations of LMs’ linguistic capacity by applying experimental paradigms. They examined the difficulties that a neural processor might undergo during sentence processing with a set of stimuli designed for a targeted linguistic experiment on the human processor, often using long-distance dependency (LDD)^1^ phenomena such as filler–gap dependencies (Wilcox et al., 2018; Chaves, 2020), subject–verb agreement (Linzen et al., 2016; Gulordava et al., 2018; Goldberg, 2019; Jawahar et al., 2019), and the licensing of negative polarity items (henceforth, NPIs; Jumelet and Hupkes, 2018; Warstadt et al., 2019; Jumelet et al., 2021). LDDs serve as a useful testbed for these purposes because the integration of long-distance or non-adjacent words or phrases is considered the hallmark of any good language processor like adult speakers. LDDs require an understanding of the hierarchical sentence structure, as opposed to a linear string of words. This line of research has shown that state-of-the-art LMs such as Bidirectional Encoder Representations from Transformers (BERT; Devlin et al., 2019) and long short-term memory (LSTM; Hochreiter and Schmidhuber, 1997; Jozefowicz et al., 2016) are highly capable of processing several LDD tasks, suggesting they have significant grammatical sensitivity to hierarchical structures.

Drawing on this previous study, the present study conducts a more detailed analysis of the performance of a neural language processor, BERT. The primary purpose of this study is to compare the “surface results” in language comprehension between humans and BERT. To that end, we used NPI phenomena as a test case, namely, whether BERT processes NPIs in the same way as humans do in online sentence comprehension. NPIs are one of the LDDs that require the use of highly complex processing algorithms since the dependency between an NPI and its licensor can be defined not only by syntactic constraints but also by complex semantic features. We examine how BERT processes NPIs while varying the semantic and syntactic conditions in the stimuli just as we conduct any psycholinguistic experiment to investigate the underlying mechanisms of human language processing. We focus on not only successful but also unsuccessful processing patterns (i.e., grammatical illusion). We compare the results with those obtained from human NPI processing and discuss the similarities and differences between human and neural language processors. This study also intends to examine how viable LMs are as a new research tool and object of language research in general.

### 1.1. NPIs in linguistics and psycholinguistics

Negative polarity items such as *ever* pose a critical challenge to language processors. When encountering an NPI, a successful processor integrates it with a preceding non-adjacent word or licensor to make sense of the sentence. Once a licensor is identified, the processor checks whether the syntactic and semantic relationships of the two linguistic elements accord with grammatical constraints imposed on the dependency between them. For example, the NPI *ever* is semantically dependent on or licensed by the occurrence of a so-called negative word that precedes the NPI such as *no*, as illustrated in (1a). The positive word *some* makes the sentence ungrammatical, as in (1b). In addition, syntactically, the negative licensor *no*, and the NPI *ever* are syntactically required to occur within the same clause boundary as shown in (2a). A simple linear precedence relationship between a licensor and an NPI does not suffice to make their long-distance relationship legitimate, as shown in (2b), i.e., *no* occurring within the embedded clause, while *ever* is outside of it, i.e., *[_CL1_The politicians [_CL2_who no protesters met] *ever* supported the bill].

1. a. No politicians *ever* supported the bill.
  b. *Some politicians *ever* supported the bill.
2. a. No politicians who the protesters met *ever* supported the bill.
  b. *The politicians who no protesters met *ever* supported the bill.

A body of psycholinguistic research has investigated NPI processing in the context of online sentence comprehension (Xiang et al., 2009, 2013; Parker and Phillips, 2016, among many others). It is an intriguing topic for psycholinguists, particularly because it has revealed not only the human processor’s syntactic accuracy but also its fallibility, called *grammatical illusion*. Studies have shown that human processors are generally successful in processing the dependency between an NPI and a licensor but sometimes mistakenly accept a wrong licensor, such as *no* in the embedded clause in (2b), as a legitimate one for an NPI in the main clause, which violates the licensing constraint, i.e., NPI illusion. By observing when the usually effective processor is led into error, we can induce the linguistic information that the processor exploits and can also reconstruct the mechanisms by which the processor works. Thus, processing failures often provide useful information for studying the mechanisms underlying language processors.

Negative polarity items have received a significant amount of attention from researchers in formal semantics, as the semantic properties of grammatical NPI licensors are difficult to pin down. It has been shown that they tend to involve complex semantic relationships beyond simply being “negative.” Semanticists have proposed four criteria that characterize the semantics of legitimate NPI licensors, as illustrated in Table 1.

**Table 1:** Semantic properties of NPI licensing contexts.

Type	Example	Semantic properties		
		Anti-additive	Downward-entailing	Non-veridical
Negative	**No** students *ever* liked linguistics.	+	+	+
	**Few** students *ever* liked linguistics.	−	+	+
Zero-negative	Has John *ever* liked linguistics?	−	−	+
	**Only** John *ever* liked linguistics.	−	−	−

Negative licensors, such as *no* and *few*, are categorized by the semantic property called downward entailment (Ladusaw, 1979), which refers to a semantic relationship from a set to a subset such that, given *semantics* is a subset of *linguistics*, the sentence *No/Few students liked linguistics* entails *No/Few students liked semantics*, i.e., X ⊆ Y, *f*(x) ⊆ *f*(y). Classic negation (or negative quantifiers) like *no* is further distinguished from minimal negation such as *few* in that the former is not only downward-entailing but also anti-additive, i.e., *f*(X) ∪ *f*(Y) = *f*(X) ∩ *f*(Y), while the latter is not (Zwarts, 1996). The occurrence of NPIs can also be licensed by zero-negative but non-veridical expressions such as questions, imperatives, and modal expressions, i.e., a propositional operator *F* is veridical iff *F*p → p. Non-veridicality is proposed to be the minimal semantic requirement to license an NPI (Giannakidou, 1998). However, research has shown that even some veridical expressions such as *only* justify the occurrence of a certain class of NPIs. It has been argued that such a veridical context does not semantically license an NPI in a strict sense but at least “rescues” the NPI through pragmatic inference such that *only John* can be interpreted with a negative word such as “no one but John” (Giannakidou, 2006).

What is crucial about the four semantic categories in terms of NPI licensing is that the semantic properties are related to the gradience or scale of licensing strengths (Zwarts, 1996; Giannakidou, 1997). For example, classic negation and negative quantifiers associated with all three semantic properties are stronger licensors than minimal negation associated with only two properties. Similarly, minimal negation is stronger than merely non-veridical contexts in its licensing strength. Finally, the veridical contexts, which only globally support NPIs, are considered the weakest licensors. This theoretical proposal has led to the hypothesis that the strength of the licensors modulates the integration between an NPI and its licensor (refer to Giannakidou and Etxeberria, 2018, for a review). Namely, a stronger licensor better integrates with an NPI. Chatzikontantinou et al. (2015) empirically examined the effect of the licensing strengths based on a sentence judgment experiment. They hypothesized that stronger licensors lead to more positive acceptability ratings on sentences that include an NPI. The results revealed significant differences in acceptability ratings depending on the choice of licensors, i.e., [*no* (classic negation) > *very few* (minimal negation)] > *only* (zero negation), confirming the theoretical proposal. The results suggest that the degree of negativity is a significant factor that can modulate human NPI processing.

### 1.2. The present study

Some recent studies have examined NPI licensing in neural language models. For example, Warstadt et al. (2019) performed a general evaluation of a neural language model’s performance on NPI processing and showed that the model exhibits meaningful sensitivity to the combination of an NPI and its licensing contexts. Jumelet et al. (2021) also showed that neural models have the semantic sensitivity of distinguishing downward-entailing licensors from others. In this context, the present study investigates further details in a deep neural model’s syntactic and semantic sensitivity to NPIs using a psycholinguistic paradigm. We conduct experiments with one of the most recent and successful neural language models, BERT (Devlin et al., 2019). In Experiment 1, we investigate whether BERT can successfully process the syntactic constraints of NPI licensing introduced earlier. More specifically, we study whether it can discriminate between syntactically correct and incorrect sites of a licensor in a hierarchical sentence structure, i.e., within vs. across clause boundaries. In addition, we examine whether it can be led to grammatical illusion by a licensor occurring in an incorrect site. This is particularly interesting because the phenomenon is known to occur in human sentence processing only in a transient manner and disappears after the human processor is allowed sufficient processing time. In Experiment 2, we investigate whether BERT is also sensitive to the semantic components of NPI licensors and their licensing strengths, i.e., *no* > *few* > *only*, as observed in human speakers. We assess the model’s performance based on the cloze test approach (Goldberg, 2019) and the measure of surprisal estimation (Hale, 2001; Levy, 2008; Chaves, 2020; Chaves and Richter, 2021) used in previous studies. Our study observes the “behavior” of a neural language model with more rigorously designed experiments from a psycholinguistic perspective. We expect this study to enhance our understanding of the mechanisms underlying an up-to-date neural language model, particularly with respect to NPI licensing.

## 2. Experiment 1: Syntactic licensing and grammatical illusion

In this experiment, we examined the syntactic knowledge of a neural language model BERT with respect to NPI processing and the possibility of its processing failure as well. Namely, we tested whether the model captures a structurally hierarchical relationship between an NPI and its licensor as opposed to a simple linear precedence relationship and also whether it is susceptible to the erroneous licensing of a syntactically illicit licensor that holds only a linear relationship, i.e., a phenomenon called NPI illusion in psycholinguistics.

### 2.1. Method

#### 2.1.1. Materials

We adapted the sentence stimuli used in Xiang et al. (2009) for current purposes. Their material is designed for a psycholinguistic experiment and the structural position and the presence of a potential licensor are rigorously manipulated, as illustrated in Table 2. Namely, in the *licit licensor* condition, the licensor *no* of the NPI *ever* occurs in the matrix clause, as does the NPI, conforming to the syntactic licensing constraint. In the *illusory licensor* condition, the potential licensor *no* and the NPI belong to different clauses, i.e., the embedded and the main clause, respectively, violating the syntactic constraint but only holding a linear precedence relationship. It is so named because research has shown that it can be mistakenly processed as a legitimate licensor, given an NPI. In the *no licensor* condition, a licensor is absent. All the other settings are kept the same across the triplet. The total number of words and the position of an NPI are the same within each set of three sentences, e.g., 19 words with *ever* at 14th in all three sentences shown in Table 2. The material included 150 sets of such triplets, i.e., 450 sentences in total. In the actual implementation of the stimuli, the NPI *ever* is masked to use the cloze test method (Goldberg, 2019). The procedure of data extraction using masks is explained in the next section.

**Table 2:** Three conditions in Experiment 1 and example stimuli (from Xiang et al., 2009).

Condition	Sentence example
Licit licensor	No scandals that the prominent politicians have been willing to discuss publicly have **ever** generated a large public outcry.
Illusory licensor	*The scandals that no prominent politicians have been willing to discuss publicly have **ever** generated a large public outcry.
No licensor	*The scandals that the prominent politicians have been willing to discuss publicly have **ever** generated a large public outcry.

#### 2.1.2. Modeling procedure and analysis

As alluded to earlier, we used the cloze test method, following Goldberg (2019). The slot in each sentence from which word probabilities are extracted was masked. For example, the position of the NPI *ever* was masked from the stimuli in Table 2, i.e., *… willing to discuss publicly have [MASK] generated a large public outcry*. We extracted the softmax values or probabilities of *ever* in the masked position from all three conditions and then converted them to surprisal values (Wilcox et al., 2018; Chaves and Richter, 2021). Surprisal is the negative log probability of a word given a context and is shown to correlate with the degree of cognitive effort the human processor exerts to process a word, i.e., as a proxy for processing difficulty (Hale, 2001; Levy, 2008). Namely, a word with a low probability has a high surprisal value, indicating greater cognitive effort. Following Chaves and Richter (2021), we estimated the surprisal of a word by computing the negative log probability based on the softmax values before consuming the word, given all the other words in the sentence. We analyzed the results using one-way ANOVA and performed Tukey tests for pairwise comparisons between conditions.

In the actual implementation of neural language experiments based on BERT, the goodness of the licensing relationship can be estimated by word probabilities of licensors rather than those of NPIs (e.g., Warstadt et al., 2019), due to BERT’s bidirectional representations of a sentence. Thus, we test our hypothesis by examining the probabilities at both the licensor’s and the NPI’s positions. For the purpose of this experiment, however, masking licensors may be problematic since there is no licensor to be masked in the no licensor condition. We examine our hypothesis in a slightly modified setting as follows. We first measured the surprisal of *no* at the licensor position in the main clause (licit licensor) and in the embedded clause (illusory licensor), respectively, and compared them to examine whether the neural model discriminates between the grammatical and ungrammatical positions of licensors. In addition, we compute the surprisal of the definite article (*the*) at the same positions as an estimate of a no-licensor condition at each site, e.g., *[No/The]_main_ scandals that [no/the]_embedded_ prominent politicians have been …*. The surprisal of *no* and *the* in the main and the embedded clause are compared, respectively, to examine whether the model discriminates between good and bad (or no) licensors in the syntactically correct and incorrect sites, respectively. We performed a two-way ANOVA to analyze the effect of syntactic position (main and embedded) and licensor (*no* and *the*) on surprisal and Tukey tests for pairwise comparisons.

We analyzed the two sets of data collected from NPI positions and licensor positions. But note that the investigation based on the NPI positions introduced earlier can straightforwardly serve the purpose of this experiment, directly comparing the three conditions in Table 2, while the analysis from the licensor positions can be useful in confirming the results from the NPI positions, e.g., comparing *no* between the main and embedded clauses, and also in examining potentially different behavior in the main and embedded clauses, respectively. In this experiment, we used a BERT base with 110 million learning parameters, 768 hidden layers, 12 transformer blocks, and a maximum of 512-word context windows.

### 2.2. Results and discussion

**Figure 1:**
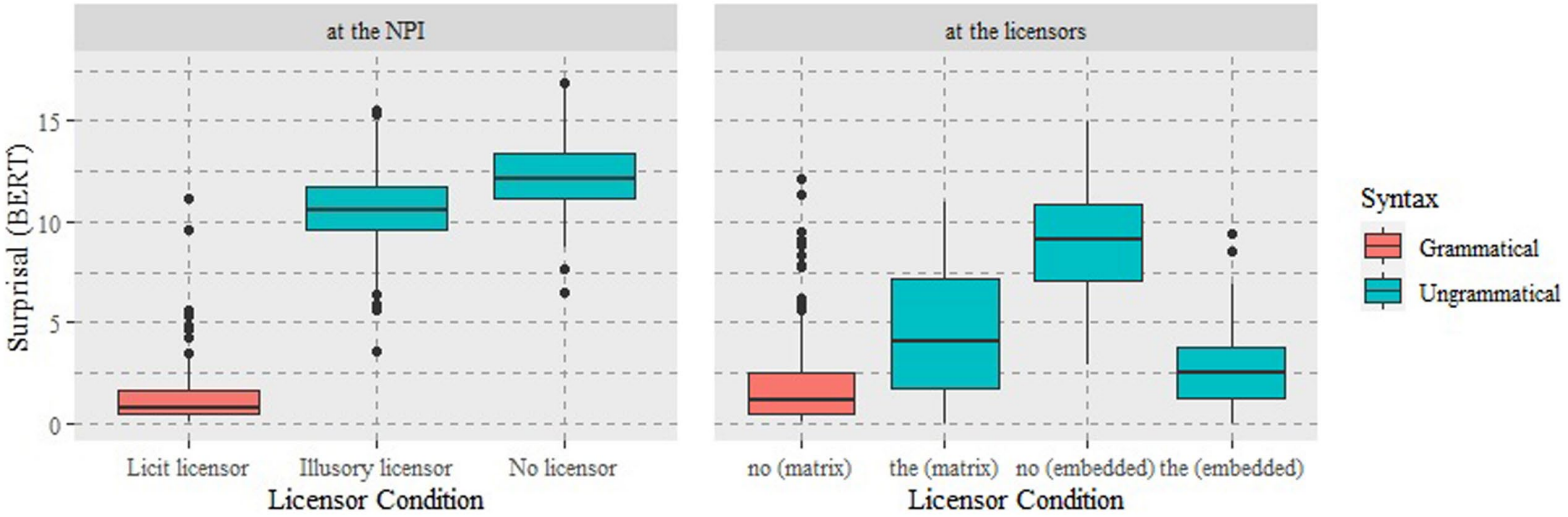
The distribution of surprisal at the NPI and at the licensor positions in Experiment 1.

As illustrated in Figure 1, the results based on surprisal *at the NPI* show that the mean surprisal on the NPI *ever* was dramatically lower in the *licit* licensor condition (*M* = 1.30, SD = 1.49) than in the other two *illusory* and *no-licensor* conditions. Note that only the *licit* licensor condition is a grammatical condition. Between the latter two, the mean surprisal of the no-licensor condition was higher (*M* = 12.2, SD = 1.80) than that of the illusory licensor condition (*M* = 10.6, SD = 1.89). One-way ANOVA was performed to compare the effect of licensor conditions on surprisal values, and a statistically significant difference in surprisal was noted between at least two groups (*F*(2, 447) = 1728.9, *p* < 0.001). The post hoc Tukey test for multiple comparisons revealed that the mean value of surprisal was significantly different in all pairwise comparisons, i.e., between licit and illusory conditions (*p* < 0.001) between illusory and no-licensor conditions (*p* < 0.001), and between licit and no-licensor conditions (*p* < 0.001).

The results based on surprisal *at the licensor* show that the mean surprisal of *no* in the main clause (licit licensor) (*M* = 2.05, SD = 2.36) was lower than that of *the* in the same clause (*M* = 4.45, SD = 2.96) and that of *no* in the embedded clause (illusory licensor) (*M* = 9.03, SD = 2.66), as expected. Within the embedded clause, the mean surprisal of *no* was higher than that of *the* (*M* = 2.62, SD = 1.72), as opposed to the result within the main clause. The results of two-way ANOVA revealed statistically significant main effects of syntactic position (*p* < 0.001) and licensor (*p* < 0.001) as well as a significant interaction between them (*F*(3, 596) = 246.8, *p* < 0.001). A post hoc analysis on the nature of interaction showed that the surprisal of *no* is lower than that of *the* within a main clause, but the surprisal of *no* is higher than that of *the* within an embedded clause. The post hoc Tukey test showed there is a statistically significant difference between *no* in the main and *no* in the embedded clause (*p* < 0.001), between *no* and *the* within the main clause (*p* < 0.001), and between *no* and *the* within the embedded clause (*p* < 0.001).

Overall, the results showed that the neural model BERT is highly sensitive to the syntactic licensing condition for the NPI *ever*. The significant difference between the *licit* and *no* licensor conditions suggests that the model “understands” the long-distance dependency between the NPI and its licensor in line with human processors. More importantly, the significant difference between the *licit* and *illusory* licensor conditions suggests that the model discriminates between syntactically correct and only linearly preceding but hierarchically incorrect licensors. In addition, the opposite patterns of *no* and *the* between the main and embedded clauses, i.e., *no* is “better” than *the* within the main clause but *the* is better than *no* within the embedded clause, further support the syntactic sensitivity of the neural language model. Namely, the result suggests that when an NPI belongs to the main clause, the model predicts a legitimate licensor occurs in the main-clause position (lower surprisal) but not in the embedded-clause position (higher surprisal). This indicates that BERT has substantial grammar-like knowledge required to process the long-distance relationship between an NPI and a licensor. Finally, the difference between the *illusory* and *no* licensor conditions suggests that BERT cannot completely reject a structurally illicit negative (or illusory) word as a licensor, suggesting that BERT may be susceptible to grammatical illusion to some degree, analogous to the fallibility of the human processor shown in psycholinguistic experiments. This experiment demonstrates that BERT’s syntactic processing with respect to NPI licensing is substantially similar to humans not only in its capability but also in its fallibility.^2^

## 3. Experiment 2: Semantics and scale of licensing strength

As noted in the introduction, NPI licensors are semantically highly complex and varied. Semanticists have shown that the contexts in which NPIs occur cannot be generalized simply as being negative, but differ in fine-grained semantic components, such as anti-additivity, downward entailment, and (non-)veridicality. Crucially, it is hypothesized in both theoretical and psycholinguistic approaches to NPIs that how many of the semantic components a licensor has in meaning is closely related to their scale of negativity and, thus, to their licensing strengths. In Experiment 2, we examined whether the deep neural model BERT can also capture the semantic differences between licensors of different strengths when an NPI *ever* is present in a sentence. Given that Experiment 1 showed BERT to be syntactically highly sensitive, the results of the present experiment will reveal whether it is also semantically as sensitive as human processors regarding NPI licensing.

### 3.1. Method

#### 3.1.1. Materials

Of the four semantic categories of licensors introduced in Table 1, we used three conditions in this experiment, i.e., “no” *classic negation* (anti-additive, downward entailing and non-veridical), “few” *minimal negation* (downward entailing and non-veridical), and “only” *veridical* (zero-negative and veridical) conditions. The zero-negative and non-veridical context was excluded because it can only be realized in specific syntactic structures such as questions (*Have you ever been to Europe?*), imperatives (*Do not ever say that*), and modals (*No one could have ever predicted these results*). In order to compare the licensors’ semantic effects more accurately, the sentences with semantically different licensors should be controlled for their syntactic structure since Experiment 1 showed that syntactic structure significantly modulates BERT’s NPI processing. We chose the three conditions in which the semantic licensors can replace each other at the sentence-initial position while their overall sentence structures are kept constant. In addition to the three potentially possible licensors, we added the fourth condition that contains a semantically impossible licensor *the* as a baseline. We adapted the sentences used for Experiment 1 for the present purposes. We removed the embedded clause from the original sentences as they are irrelevant to our semantic hypothesis in this experiment, as illustrated in Table 3. All others were kept constant.

**Table 3:** Four conditions in Experiment 2 and example stimuli (adapted from Xiang et al., 2009).

	Condition	Licensor	Sentence example
Semantically possible	Classic negation	*no*	{No/Few/Only/The} scandals have **ever** generated a large public outcry.
	Minimal negation	*few*	
	Zero-negation	*only*	
Semantically impossible	Non-negation	*the*	

#### 3.1.2. Modeling procedure and analysis

As with Experiment 1, we used the cloze test method and surprisal estimation based on BERT-base. As before, we measured the surprisal of both the NPI (*ever*) and the licensors (*no/few/only/the*) in all four conditions. All other settings were kept constant. The masks were placed at the NPI position for the former setting, e.g., *No/few/only/the scandals have [MASK] generated a large public outcry*, while they were in the sentence-initial position for the latter, e.g., *[MASK] scandals have ever generated a large public outcry*. We analyzed the two sets of data, i.e., at the NPI and the licensor position, respectively, using ANOVA and performed Tukey post hoc comparisons as before.

### 3.2. Results and discussion

The surprisal extracted at the NPI (*ever*) was analyzed by a one-way ANOVA. There was a statistically significant difference between conditions (*F*(3,596) = 661.08, *p* < 0.001). A Tukey post hoc test revealed that there is a significant difference between *no* and *few*, between *no* and *the* as well as between *few* and *the* (all three, *p* < 0.001). But the difference between *few* and *only* was not significant (*p* = 0.66). The result based on the surprisal of the licensors showed there is again a statistically significant difference between conditions (*F*(3,596) = 1909.09, *p* < 0.001). A Tukey post hoc test showed there is a significant difference in all pairwise comparisons, respectively (all, *p* < 0.001).

**Figure 2:**
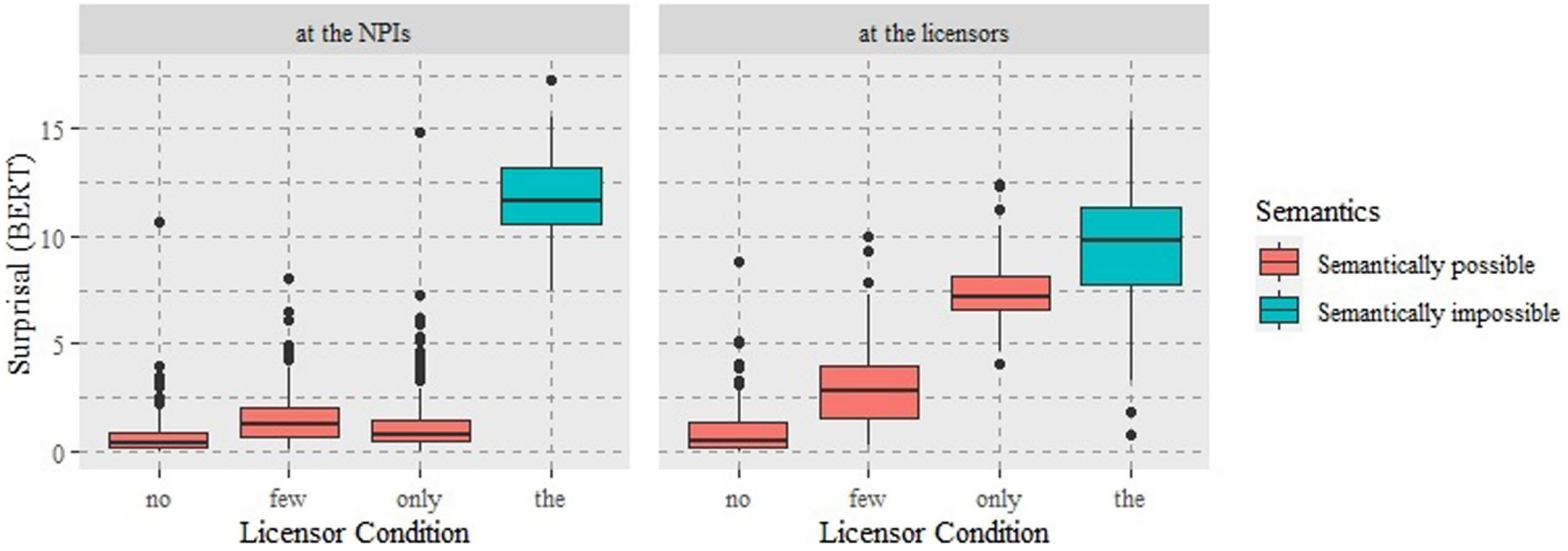
The distribution of surprisal at the NPI and at the licensor positions in Experiment 2.

As illustrated in Figure 2, the neural model clearly distinguishes the semantically possible licensors from the impossible ones. The mean surprisal of *no* in the anti-additive, downward entailing, and non-veridical condition, which is often referred to as classic negation, was the lowest in both mask settings (*M* = 0.72, SD = 1.10 at the NPI; *M* = 0.93, SD = 1.15 at the licensor). The mean surprisal of *few* in the downward entailing and non-veridical condition, which is often referred to as minimal negation, was the second lowest in both mask settings (*M* = 1.53, SD = 1.30 at the NPI; *M* = 2.95, SD = 1.78 at the licensor). The mean surprisal of *the*, semantically impossible licensor, as a baseline was, as expected, the highest in both mask settings (*M* = 11.9, SD = 1.79 at the NPI; *M* = 9.53, SD = 2.76 at the licensor). By contrast, the mean surprisal of zero-negative and veridical *only* differed between the two mask settings, i.e., lower (*M* = 1.33, SD = 1.73) at the NPI but higher (*M* = 7.42, SD = 1.43) at the licensor, compared to that of *few* at the respective setting.

To sum up, we found consistent patterns in mean surprisal values between the classic negation *no*, the minimal negation *few*, and the semantically impossible licensor *the* regardless of specific mask settings. However, the computational evaluation of the relationship between *ever* and *only* differed depending on where the surprisal was measured. Namely, the hypothesis on scalar negativity and licensing strengths was fully borne out when surprisal was measured at the licensor and was partially confirmed when surprisal was measured at the NPI.

Despite the inconsistencies found in the case of *only*, the results for *no*, *few*, and *the* suggest that the neural model BERT can process the semantics of negativity and its relationship with NPIs, similar to what was found in psycholinguistic experiments with humans. With respect to semantic entailment relationships, the result suggests that BERT significantly distinguishes anti-additive and downward-entailing licensors such as *no* from merely downward-entailing ones like *few*.^3^ The different results for *only* between the two mask settings, we suspect, can be attributed to their semantic and pragmatic complexity. As introduced earlier, *only* is veridical, as opposed to the other two, *no* and *few*. Semanticists have shown that, in principle, veridical contexts cannot license NPIs. Giannakidou (2006) suggested that what *only* does with an NPI is not genuine semantic licensing but should be referred to as some type of “rescuing” through pragmatic inference. In addition, computational studies attempted to examine the pragmatic ability of BERT. Some studies showed neural language models are capable of pragmatic inferences (Warstadt et al., 2019; Jeretic et al., 2020; Pandia et al., 2021), while others argued BERT is simply a “stochastic parrot” learning the surface distribution of linguistic forms and has no access to the communicative intent of messages (Bender and Koller, 2020). In this context, we can only speculate about why BERT exhibited the result for “only” against our prediction. It is possible that BERT simply fails to capture the statistical regularity of co-occurrences of *only* and *ever* or that BERT fails to correctly narrow down the use of *only* as an NPI licensor since *only* has many different meanings and functions, e.g., domain restrictor, superlative, depending on context. The semantic and pragmatic property of *only* and the potential limitations of the model seem to lead to shaky results in modeling its semantics with BERT, while negative licensors can semantically and directly license an NPI to produce consistent results.

Note that we checked the possibility that the difference in *only* between two mask settings arose from the asymmetric conditional probabilities of occurrences between *only* and *ever* in texts in general. The surprisal of *only* was computed when *ever* was already present; the surprisal of *ever* was computed when *only* was already present. It is possible BERT was affected by relational word frequency differences, as the model was trained on actual texts. To verify this possibility, we randomly sampled 1,000 sentences that contained “*only* N” and *ever*, respectively, from the Corpus of Contemporary American English (Davies, 2008-) and found their conditional probabilities are more or less balanced, e.g., P(*ever*|*only*) = 2.8% and P(*only*|*ever*) = 2.8%. In addition, Li et al. (2021) recently showed by a layerwise model analysis that the effect of frequency information is strong only in the lower layers of Transformer language models like BERT but eventually decreases in the upper layers. Thus, we exclude the possibility that the unequal results for *only* in the two settings are simply an artifact of word frequencies. This discrepancy seems to require further research.

The last thing to note is that the meaning of word probabilities slightly differs depending on mask positions. In other words, when measuring them at the NPI position, we ask the model how predictable a specific NPI is with potentially (im)possible licensors being already present; at the licensor position, we ask the model how predictable a specific licensor is when an NPI is already given in a sentence. Note also that for a sentence to be well-formed, the NPI *ever* is not required even when potential licensors are present (e.g., *No scandals have (ever) generated a public outcry*), but the licensors are required when the NPI *ever* is present (e.g., *Scandals have ever generated a public outcry). Thus, we suspect that the results at the NPI position reflect the model’s decision on whether the NPI *ever* is possible or not when potentially possible or impossible licensors are present, e.g., a largely binary result (Figure 2, left), while those at the licensors can be relatively sensitive to which licensor has a better fit with the given NPI, i.e., a gradient result (Figure 2, right). Our results suggest that an examination of semantic fit or licensing strength between an NPI and a licensor can be better measured at the licensors when using a bidirectional model.

## 4. General discussion

We examined whether the deep neural model, BERT, can capture the highly complex syntactic and semantic constraints of NPI licensing and whether the results are similar to those observed with human subjects. We found in Experiment 1 that BERT is a highly sensitive syntactic processor. BERT discriminates between the presence and absence of a licensor in a grammatical position and can discern between a licensor occurring in a grammatical position and one occurring in an ungrammatical position in a hierarchical syntactic structure. Another intriguing result is that BERT considers illicit licensors in a syntactically wrong position better than having no licensors at all. This constitutes the mistaken acceptance of a syntactically illicit licensor, which occurs in sentences that humans also have grammatical illusions (Xiang et al., 2009; Parker and Phillips, 2016). In Experiment 2, we found mixed results with regard to BERT’s semantic knowledge. BERT clearly distinguished semantically possible licensors from impossible ones and was also shown to be highly sensitive to the differences in possible licensors as to semantic entailment, i.e., classic and minimal negations and non-veridical *only*, replicating the results of human judgment regarding scalar negativity and licensing strengths (Zwarts, 1996; Giannakidou, 1997; Xiang et al., 2013; Chatzikontantinou et al., 2015). The results suggest that BERT can process semantic features to a significant degree. This experiment also showed that specific implementational settings, such as mask positions, might modulate the results when working with a bidirectional Transformer model. The results of these two experiments suggest overall that the neural language model, BERT, is highly sensitive, both syntactically and semantically, in processing the long-distance dependency between an NPI and its licensor. Their processing patterns and sensitivities are shown to be very close to humans, suggesting their role as research tools and objects in psycholinguistics.

The results of our study are in line with those of previous research that demonstrated BERT’s success in syntactic processing (Marvin and Linzen, 2018; Goldberg, 2019; Van Schijndel et al., 2019; Warstadt et al., 2019; Chaves and Richter, 2021, among others). Our results are particularly interesting in that we demonstrated not only that BERT is highly capable of syntactic processing but also that it may be prone to an error similar to the grammatical illusion that was shown to occur in NPI processing experiments with humans. NPI illusion is reported to be transient and is not observed when speakers are allowed enough time to process the sentence. In our experiment, BERT showed an intermediate surprisal value higher than that of a perfect licensor and lower than that of an impossible licensor. It seems worth investigating how the transiency and the medium degree of surprisal can be analogous to each other in future research.^4^

In addition, the results advance our understanding of BERT’s capability in semantic processing. There have been only relatively rare and mixed results about BERT’s semantic sensitivity in the literature. For example, Ettinger’s (2020) diagnostics demonstrated that BERT does not perform well in processing negation and inference but is good at retrieving noun hypernyms. Tenney et al. (2019) showed that BERT could encode semantic role information in sentence processing. Jumelet et al. (2021) showed using an LSTM model that the model has sufficient semantic knowledge to distinguish between downward- and upward-entailing lexical items. Our study further showed that BERT could make an even more fine-grained semantic distinction between classic and minimal negation, even though both are downward entailing. Overall, this study suggests that a deep neural model may “act” like a human processor in both syntactic and semantic language processing and can be used for linguistic and psycholinguistic research as a near-human language processor or learner.

Note, however, that we do not argue that BERT and humans process language in exactly the same way in every detail. There are known fundamental differences in their mechanisms and in the measures of the processing difficulty. What characterizes human sentence comprehension is incrementality. The human processor takes a sentence incrementally from the first word to the last one, i.e., unidirectional. BERT, however, is bidirectional, meaning it can read a sentence from both ends simultaneously. Thus, in human sentence comprehension studies, the surprisal at a target word is estimated based only on what the processor has already experienced. However, the surprisal estimation using BERT is based on the sum of word probabilities for the sentence as a whole except the target word, and it includes the words that *follow* the target word since the model takes into account the backward direction. Thus, we may not equate the surprisal values in human studies with those in BERT-based ones. However, at the performance level, BERT may have reached the same goals along different routes. Our results suggest that BERT exhibits the knowledge necessary for processing linguistic input and returns results analogous to what humans show in language comprehension. Our results also suggest BERT can be a testable toolset for linguists and psycholinguists that can simulate human language processing.

Finally, the present study showed that BERT can be a useful tool for comparing different languages with different word orders. For example, with respect to NPIs, some languages such as English require retrospective licensing, i.e., a licensor occurring before an NPI, while others such as Japanese, Korean, and Turkish require prospective licensing, i.e., a licensor occurring after an NPI. In our experiments, we measured surprisal at two different sites, i.e., at the NPI position and the licensor position, taking advantage of BERT’s bidirectionality. The fact that we found similar results in two mask positions suggests BERT can be used in making crosslinguistic comparisons such as between an NPI after a licensor in English and an NPI before a licensor in Korean, everything else being equal. However, the bidirectionality of BERT may also be a disadvantage in investigating a linguistic phenomenon strongly driven by the unidirectional, incremental, and predictive nature of human sentence processing. For example, upon the human processor’s early encountering of an NPI in prospective licensing languages, the occurrence of a licensor is strongly expected and the illusion effect is also shown to be relatively robust (Yanilmaz and Drury, 2018; Yun et al., 2018). By contrast, in retrospective licensing languages, an NPI may be little or not even expected by a preceding negative word. When one attempts to emulate any processing features strongly characterized by the incremental and unidirectional nature of NPIs, sequential models seem to serve the purpose better.

## Data availability statement

The original contributions presented in the study are included in the article/Supplementary material, further inquiries can be directed to the corresponding authors.

## Author contributions

US, SS, and EY contributed to conception and design of the study. US conducted the experiments and performed statistical analysis. EY wrote the manuscript. All authors contributed to manuscript revision, read, and approved the submitted version.

## Funding

This work was supported by the Ministry of Education of the Republic of Korea and the National Research Foundation of Korea (NRF-2020S1A5A2A03042760).

## Conflict of interest

The authors declare that the research was conducted in the absence of any commercial or financial relationships that could be construed as a potential conflict of interest.

## Publisher’s note

All claims expressed in this article are solely those of the authors and do not necessarily represent those of their affiliated organizations, or those of the publisher, the editors and the reviewers. Any product that may be evaluated in this article, or claim that may be made by its manufacturer, is not guaranteed or endorsed by the publisher.

## Footnotes

^1^We use the term “dependency” only descriptively to refer to the grammatical relationship between words that are non-adjacent or have distance in between. We do not assume any theory-dependent implications.

^2^One of our reviewers suggested including a non-NPI control for comparison that can tease apart any potential confounds, such as semantic/pragmatic effects driven by the occurrence of negative words like *no* in preceding contexts, irrespective of licensing (Steinhauer et al., 2010). We conducted the same experiments with a non-NPI adverb *often* in place of *ever* using the same experimental settings. We found the non-NPI *often* yields higher surprisal than the NPI *ever* when the potential licensor *no* is present and that the negative word *no* yields higher surprisal when the non-NPI *often* is present than when the NPI *ever* occurs instead. Overall, this additional investigation further clarifies the source of the effects we report here, reconfirming that the results are not reducible to the presence of *no* but to its licensing relationship. The details of the results are found in Appendix A included in the Supplementary Material.

^3^One of our reviewers suggested an additional experiment with another NPI *either* that has different semantic licensing requirements to test the generality of the present results. *Either* as an NPI requires anti-additivity in the semantics of its licensors and is thus termed as a strong NPI, as opposed to *ever*, i.e., a weak NPI for which downward-entailment suffices. The results confirmed our predictions, and the details are found in Appendix B in the Supplementary Material.

^4^We can only suspect that it may be due to the form of representations on which the current BERT is based. It is possible that BERT uses representations that lie somewhere between what Parker and Phillips (2016) termed the *localist* and the *holistic* representations. They suggested that encoding formats transform from the localist toward the holistic ones over the course of sentence processing and that the intrusive phenomena occur while the processor stays around the localist representations and does not fully reach the holistic ones yet. Similarly, if the current version of BERT processes language input based on such midway representations, we can expect illusory licensing to occur. This is also in line with the results of other studies, such as Chaves and Richter (2021) that showed BERT is highly successful in locally capturing number features on words but often fails to perform proper feature-matching between long-distance words, such as between a verb and its subject.
