# Supplementary material for: Investigating a neural language model’s replicability of psycholinguistic experiments: A case study of NPI licensing
Source: Front Psychol. 2023 Feb 23;14:937656. doi: 10.3389/fpsyg.2023.937656 (PMC9995786; doi:10.3389/fpsyg.2023.937656)
Supplement: Supplementary file 2 [file Data_Sheet_1.pdf]

The supplementary material to the following article:

Frontiers in Psychology
Sec. Language Sciences
Volume 14 - 2023 | doi: 10.3389/fpsyg.2023.937656

Investigating a neural language model's replicability of psycholinguistic experiments: a case study of NPI licensing

Unsub Shin, Eunkyung Yi and Sanghoun Song

Appendix A

One of our reviewers suggested to include a non-NPI control for comparison that can tease part any potential confounds driven by the early appearance of negative words irrespective of licensing mechanisms, i.e., negative cost reported in human subjects. When there are only two conditions such as the *licit licensor* and *no licensor* conditions, it may be unclear whether the difference in surprisal between the two conditions is due to the semantic and syntactic licensing effect or simply to the semantic/pragmatic effect of the negative word *no*. However, since we have the illusory condition where the negative word occurs early but in a syntactically different position compared to the licit licensor condition, we could tease apart the syntactic or positional effects beyond any semantic/pragmatic effects of the negative word *no*. As the reviewer suggested, however, we further clarified the effects with a non-NPI adverb *often* in place of *ever* in the same experimental settings. In what follows, we compare the results with the ones with the NPI *ever* in Experiment 1.

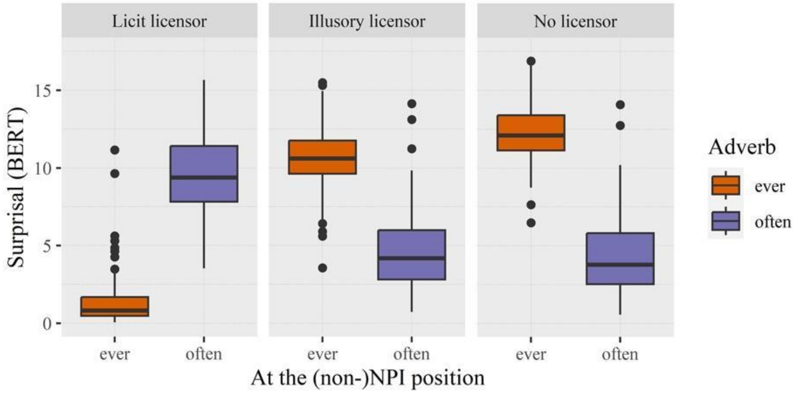

Figure A. Surprisal measured at the NPI position

As illustrated in Figure A, when measured at the NPI position (i.e., position of *ever* and *often*), the non-NPI adverb *often* yielded clearly different patterns in surprisal, compared to the NPI *ever*, as expected. The surprisal of *often* (M = 9.60, SD = 2.57) was higher than that of *ever* (M = 1.30, SD = 1.49) in the licit licensor condition, indicating an NPI is more expected. But the surprisal patterns in the

other experimental conditions were the contrary, indicating an NPI is less expected. We found in pairwise Tukey tests that the differences between *ever* and *often* are statistically significant in all three conditions ($p < .001$).

We also measured surprisal at the licensor position when *often* is present in place of *ever*. The results also confirmed our predictions. As illustrated in Figure B, the surprisal of *no* was lower (or more expected) when *ever* is present in the matrix clause (1st panel) or in the embedded clause (3rd panel). The differences were statistically significant ($p < .001$) in Tukey tests, respectively. Conversely, when *the* occurs in place of *no* in the matrix clause, its surprisal is higher (less expected) when the NPI *ever* is present than when the non-NPI control *often* is present (2nd panel). The surprisal of *the* in the embedded clause is approximately the same, whether *ever* or *often* is present (4th). The difference was non-significant.

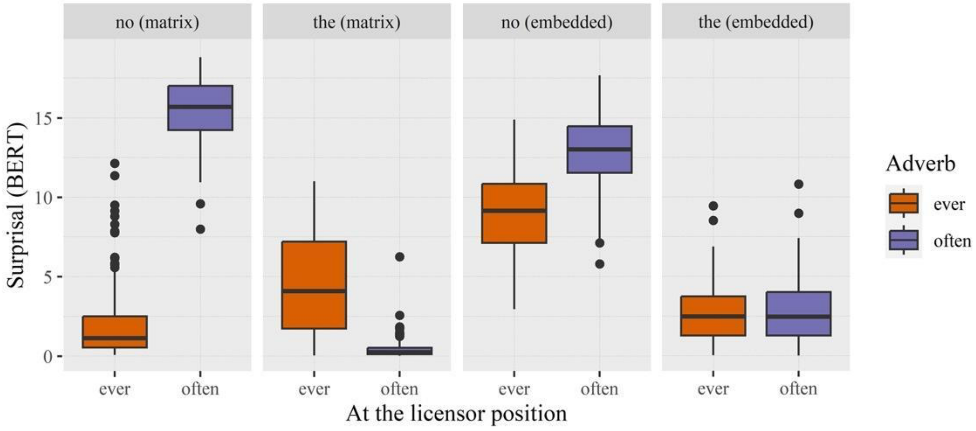

Figure B. Surprisal measured at the licensor position

It should be noted that these results reconfirm not only BERT's successful processing of NPI licensing but also the illusory licensing phenomena reported in the paper. *Ever* in general lowers surprisal or boosts expectation of the negative licensor *no*, compared to the non-NPI adverb *often* (i.e., 1st and 3rd). But, the difference between *ever* and *often* is relatively larger when *no* occurs in the matrix clause (licit licensor, 1st) than when *no* occurs in the embedded clause (illusory licensor, 3rd). In addition, the surprisal of *no* in the embedded clause leads to a significant difference between *ever* and *often* (3rd) while that of *the* in the embedded clause does not (4th). Overall, these results reconfirm the effects we reported in the paper and further clarify the source of the effects.

Appendix B

We also conducted experiments with *either*. The results with *either* can add to the generality of the paper since *either* is known to be a strong NPI as opposed to *ever* which is categorized as a weak NPI (Zwarts, 1998). We investigated the syntactic and semantic licensing conditions of *either* using the

same experimental settings used in our Experiments 1 and 2 in the paper.

It should be noted first that to the best of our knowledge, we know of no psycholinguistic studies that investigated the bahavioral patterns in the processing of *either* such as grammatical illusions and judgment variations that depend on the negativity scale. Namely, we have no psycholinguistic data to which we can compare the results of these new neural language experiments. We cannot exclude the possibility that the way humans process *either* may not be the same as the way they process *ever* since the two NPIs differ a lot not only semantically (i.e., argued-to-be possible licensors) but also syntactically (i.e., sentence-medial vs. -final positions).

That said, we simply tested whether the linguistic theories regarding the syntactic and semantic licensing conditions of *either* can be borne out with neural language models regardless of human behavior. In the first experiment, we tested the three syntactic conditions (Rullmann, 2003), i.e., licit, illusory and no licensor, using the stimuli adapted from our Experiment 1, as in (1).

(1) Example stimuli for three syntactic conditions:

a. **<u>No</u>** scandals that the prominent politicians have been willing to discuss publicly have generated a large public outcry **either**. (Licit licensor)

b. *The scandals that **<u>no</u>** prominent politicians have been willing to discuss publicly have generated a large public outcry **either**. (Illusory licensor)

c. *The scandals that **<u>the</u>** prominent politicians have been willing to discuss publicly have generated a large public outcry **either**. (No licensor)

The results revealed similar numerical patterns to those with *ever*, as illustrated in Figure A. When measured at the NPI, the mean surprisal scores tend to increase numerically from licit (i.e., grammatical) to illusory to no-licensor conditions (licit, M = 6.01, SD = 3.02; illusory, M = 10.01, SD = 3.06; no-licensor, M = 10.7, SD = 3.06). One-way ANOVA was performed to compare the effect of licensor conditions on surprisal scores, and a statistically significant difference was observed between at least two groups ($F(2, 447) = 101.12$, $p < .001$). The post hoc Tukey test for multiple comparisons revealed that the mean surprisal was significantly different between licit and illusory conditions ($p < .001$) and between licit and no-licensor conditions ($p < .001$). But, the difference between the illusory and no-licensor conditions was not statistically significant ($p = .205$), as opposed the the result with *ever* in our Experiment 1.

When measured at the licensor position, the patterns are extremely similar to those in Experiment 1. The mean surprisal of *no* is lower in the matrix clause (M = 1.35, SD = 1.72) and higher in the embedded clause (M = 4.25, SD = 2.67) than that of *the* (matrix, M = 9.49, SD = 2.44; embedded, M = 2.39, SD = 1.64). The results of two-way ANOVA revealed statistically significant main effects of syntactic position (p < . 001) and licensor (p < . 001) as well as a significant interaction between them ($F(3, 596) = 800.9$, p < .001). The post hoc Tukey test shows the surprisal difference is significant both

in the matrix and in the embedded clause ($p < .001$). The results show that when given an NPI, the licensor *no* is more expected than *the* in the matrix clause while *no* is less expected than *the* in the embedded clause.

Overall, the results of this new experiment with *either* revealed that BERT makes a robust difference between grammatical and ungrammatical positions of the licensors. But they do not confirm the possibility of grammatical illusion that was shown with *ever* in human behavioral and langauge-model studies. As noted above, it seems to need a behavioral experiment that tests whether grammatical illusion occurs in the human processing of *either*.

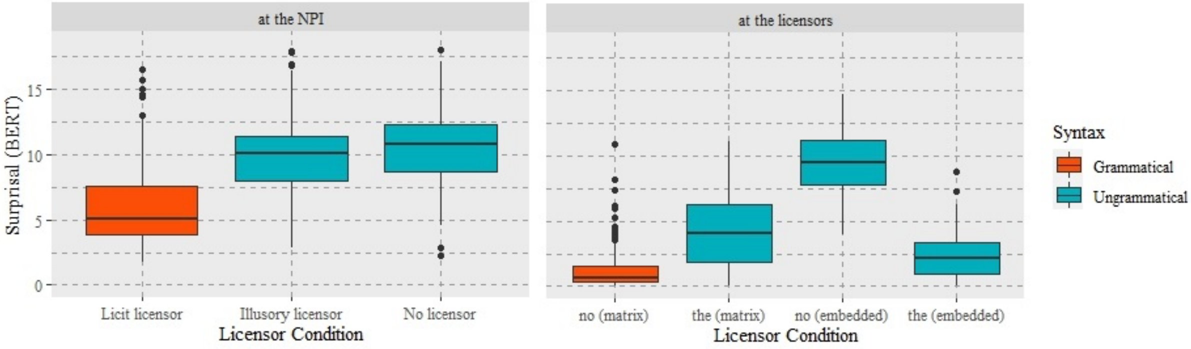

Figure A. The results of the three syntactic licensing conditions for *either*

We also conducted the same semantic experiment as Experiment 2. It should be noted, however, that the semantic conditions were modified for the following reasons: As alluded to above, the NPI *either* was initially argued to require only the strong or, namely, anti-additive licensors (e.g., *no* and *not*). More recently, researchers (e.g., Rullmann, 2003; Levinson, 2008) showed that less strong or weaker licensors such as *few* and *hardly* can also license *either*, e.g., “Few Americans have ever been to Spain. **Few** Canadians have **either**.” and “Publishers will usually reject suggestions, and writers will **hardly** accept them **either**.” To our knowledge, there is no consensus yet among semanticists as to whether weaker licensors can license the arguably strong NPI *either* (e.g., Gajewski, 2011). Thus, it is an intriguing question whether the licensing of *either* is also affected by the scale of negativity as was shown in our study with *ever*.

It should also be noted that in the at-NPIs setting, this experiment differs from Experiment 2 as to the position of *either* within a sentence. Namely, *ever* occurs sentence-medially but *either* occurs sentence-finally. This positional difference may impact the output surprisal value.

Based on the case of *ever*, we hypothesized that *either* is most easily licensed by the strong licensor such as *no* and is less so with a little less strong (and still controversial) licensor such as *few* and that the veridical *only* and the definite article *the* cannot license *either*. We conducted the experiment with the four potential licensors in three conditions, i.e., *semantically possible, no consensus* and *semantically impossible*.

(2) Example stimuli for three semantic conditions:

a. **No** scandals have generated a large public outcry **either**. (Semantically possible)

b. ?**Few** scandals have generated a large public outcry **either**. (No consensus)

c. ***Only/the** scandals have generated a large public outcry **either**. (Semantically impossible).

As illustrated in Figure B, when surprisal was extracted at the NPI (*either*), the surpisal scores tend to increase from strong to weaker licensors (*no*, $M = 5.91$, $SD = 2.39$; *few*, $M = 6.99$, $SD = 2.49$; *only*, $M = 8.53$, $SD = 2.30$; *the*, $M = 11.30$, $SD = 2.77$). The result of one-way ANOVA showed a significant difference between conditions ($F(3,596) = 129.3$, $p < .001$). A Tukey post hoc test revealed that there are significant differences across all the conditions ($p < .01$). When measured at the licensors, the surprisal of *no*, *few*, and *the* showed the same increasing pattern but that of *the* was relatively low (*no*, $M = 3.26$, $SD = 2.86$; *few*, $M = 5.98$, $SD = 2.55$; *only*, $M = 11.60$, $SD = 1.87$; *the*, $M = 4.50$, $SD = 2.53$). Again the results of one-way ANOVA showed a significant difference between conditions ($F(3,596) = 333.7$, $p < .001$). A Tukey post hoc test showed there is a significant difference in all pairwise comparisons as well ($p < .01$).

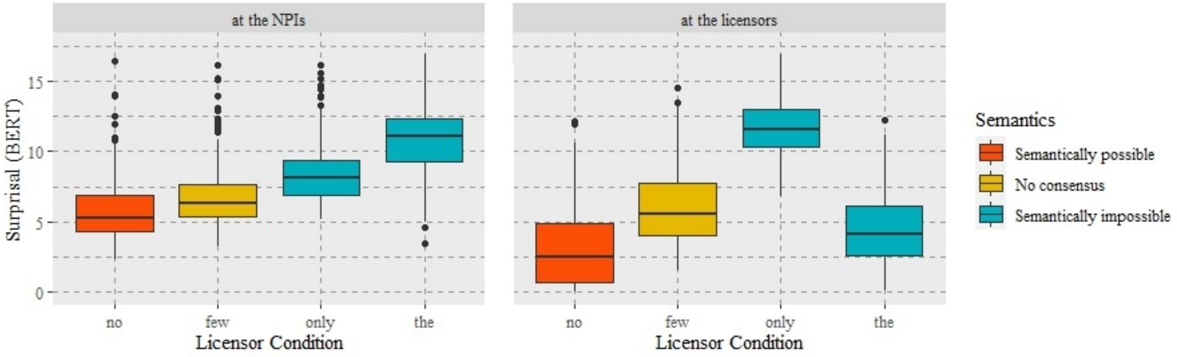

Figure B. The results of the four licensors (in three semantic conditions) for *either*

These results are quite interesting to us since they are even more nicely in line with our predictions than the results we had with *ever*. The *no*, *few* and *only* conditions yielded gradiently increasing and statistically different surprisal scores in both positional settings, which conforms to the prediction based on semantic entailments. Moreover, surprisal in the *the* condition is higher than that in the *few/only* conditions in the at-NPIs setting while it is quite low and lower than for that in the *few/only* conditions in the at-licensor setting. It conforms to our predictions. Namely, one would expect *either* when *few* is present more than when *only* is present. Also, generally, *either* is more likely to occur with the definite article *the* (apart from licensing) than with *only* in a sentence.
